# Supplementary material for: Interferometric control of magnon-induced nearly perfect absorption in cavity magnonics
Source: Nat Commun. 2021 Mar 26;12:1933. doi: 10.1038/s41467-021-22171-7 (PMC7997962; doi:10.1038/s41467-021-22171-7)
Supplement: Supplementary file 1 — Supplementary Information [file 41467_2021_22171_MOESM1_ESM.pdf]

Supplementary material for "Interferometric control of magnon-induced nearly perfect absorption in cavity Magnonics"

Rao et al

(Dated: February 13, 2021)

SUPPLEMENTARY NOTE 1: THE MAXIMAL ENERGY ABSORPTION OF A SINGLE RESONANCE

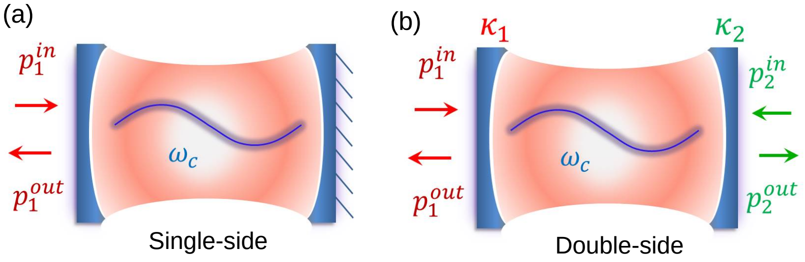

FIG. S1. (a) and (b) Illustrations of a single-port system and a double-port system.

In this section, we would generally discuss the maximal absorption rate of a single resonance. For the sake of clarity, we use a cavity resonance as an example (Fig. S1). The first case is that the cavity resonance has only one port for electromagnetic waves propagating in and out (Fig. S1 (a)). Its reflection rate can be derived from the input-output formalism:

$$S_{11} = -\frac{p_1^{out}}{p_1^{in}} = 1 + \frac{\kappa}{i(\omega-\omega_c) - (\frac{\kappa}{2}+\gamma)} \tag{S1}$$

where κ is the coupling strength between the cavity resonance and the extra-cavity photon bath. ω_c and γ respectively represent the mode frequency and its intrinsic damping rate. When $\omega = \omega_c$, the reflection of the cavity resonance reaches the minimal value. Under this condition, the absorption rate of the cavity resonance reaches the maximum, which is:

$$Abs.(\omega) = 1 - |S_{11}|^2 = \frac{2\kappa\gamma}{(\kappa/2+\gamma)^2} \tag{S2}$$

From this equation, the perfect absorption (100%) can be achieved, as long as the condition of $\kappa = 2\gamma$ is satisfied. This is the well-known critical coupling condition, which indicates the impedances at the two sides of the port are matched at the resonant frequency.

In another case, if a cavity resonance has multiple ports, for instance it has two ports (Fig. S1 (b)), we can prove that the perfect absorption for a single input signal is unachievable. According to the input-output theory, the reflection and transmission of the cavity resonance can be obtained as:

$$\begin{aligned} S_{11} &= -\frac{p_1^{out}}{p_1^{in}} = 1 + \frac{\kappa_1}{i(\omega-\omega_c) - (\frac{\kappa_1+\kappa_2}{2}+\gamma)} \\ S_{21} &= \frac{p_2^{out}}{p_1^{in}} = -\frac{\sqrt{\kappa_1\kappa_2}}{i(\omega-\omega_c) - (\frac{\kappa_1+\kappa_2}{2}+\gamma)} \end{aligned} \tag{S3}$$

If two ports are symmetric, i.e., $\kappa_1 = \kappa_2 = \kappa$, the adsorption rate at its resonant frequency is:

$$Abs.(\omega) = 1 - |S_{11}|^2 - |S_{21}|^2 = 1 - (1-\frac{\kappa}{\kappa+\gamma})^2 - (\frac{\kappa}{\kappa+\gamma})^2 \le 1 - \frac{2\kappa\gamma}{(\kappa+\gamma)^2} \tag{S4}$$

When $\kappa = \gamma$, it reaches the maximal value, i.e., 50%.

If two ports are asymmetric, i.e., $\kappa_1 \neq \kappa_2$. The absorption rate of the cavity resonance becomes:

$$Abs.(\omega) = 1 - |S_{11}|^2 - |S_{21}|^2 = \frac{2\gamma}{(\frac{\kappa_2}{2} + \gamma) + \frac{\kappa_1}{4} + \frac{(\frac{\kappa_2}{2}+\gamma)^2}{\kappa_1}} \leq \frac{2\gamma}{\kappa_2 + 2\gamma} \tag{S5}$$

When $\kappa_1 = \kappa_2 + 2\gamma$, the absorption rate reaches the maximal value, which can overpass the 50% limit as long as $\kappa_2 < 2\gamma$, but is still lower than 100%. We note that, by breaking the symmetry of two ports, the energy absorption rate from port 1 is enhanced, but it inevitable lowers the absorption rate of the input signal from port 2.

SUPPLEMENTARY NOTE 2: CAVITY MODES OF THE PLANAR CROSS CAVITY

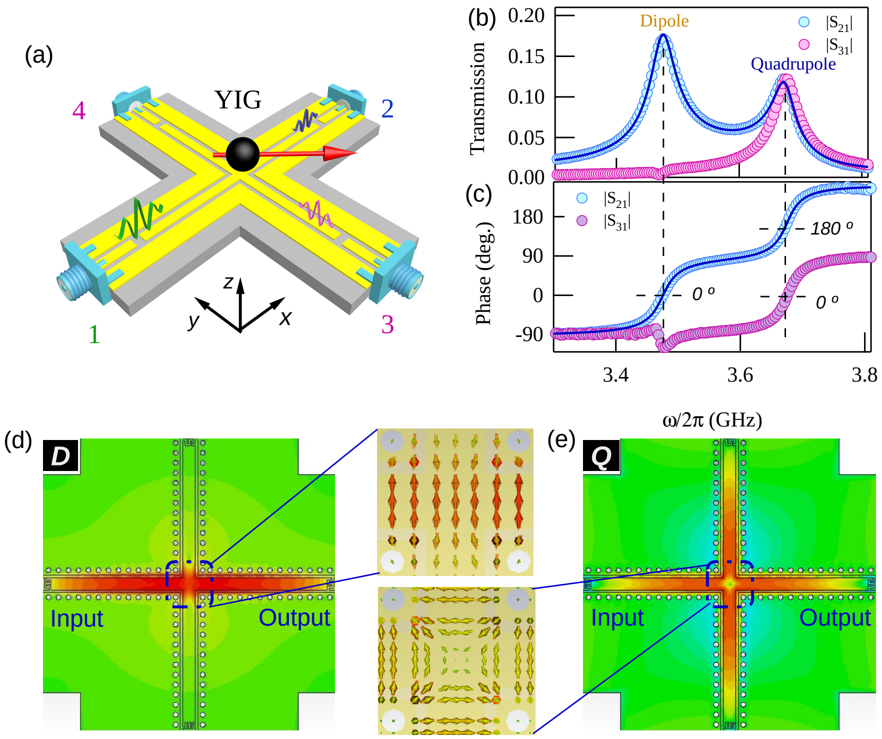

FIG. S2. (a) Schematic picture of our experimental set-up. A YIG sphere is placed at the center of the cross cavity. An external magnetic field is applied in the x-y plane to saturate the magnetization of the YIG sphere. (b) Two transmission spectra measured in either a parallel-port configuration ($1 \rightarrow 2$, $|S_{21}|$) or a perpendicular-port configuration ($1 \rightarrow 3$, $|S_{31}|$). Correspondingly, phase evolutions of these two transmission spectra are plotted in (c). Blue and red dots represent the experimental data. Solid lines are the calculation results by using Eq. (S17). (d) and (e) *Rf* h-field distributions of the D- and Q- modes in the cross cavity. In both cases, excitation signals are input from the port 1 to port 2. Two zoomed-in views respectively show the polarization of *rf* h-field at the center of cross cavity for two different modes.

The cross cavity used in our experiment is fabricated on a 0.762 mm thick RO4350B substrate, which consists of two orthogonal half-wavelength coplanar waveguide resonators with a length of 25 mm. Four feed lines (or ports) are coupled to the cross cavity via coupling gaps with a separation of 0.2 mm (Fig. S2 (a)). For such a cross cavity, its scattering parameters (S-parameter) can be measured in two different port-configurations: parallel-port ($1 \rightarrow 2$) or perpendicular-port ($1 \rightarrow 3$). Fig. S2 (b) shows the transmission spectra respectively measured at two configurations. Two resonant peaks at 3.475 GHz and 3.675 GHz can be observed from the transmission spectrum at the parallel-port configuration, but only the resonant peak at 3.675 GHz is visible at the perpendicular-port configuration. Correspondingly, phase informations of these two transmission spectra are plotted in Fig. S2 (c).

The resonant peak at 3.475 GHz indicates the parallel D-mode whose mode profile is plotted in Fig. S2 (d). We note that the parallel D-mode is a half-wavelength standing wave formed between ports 1 and 2. A zoomed-in view in Fig. S2 (d) shows the *rf* h-field polarization of the parallel D-mode at the center of cross cavity, which is linear polarized. Considering the geometric symmetry of the structure, it's straightforward to deduce that there exists another D-mode along the y-axis, which is the perpendicular D-mode. These two D-modes are degenerate and orthogonal with each other [1].

The resonant peak at 3.675 GHz is another mode of the cross cavity, named quadrupole mode (Q-mode). It's a global resonance of the whole structure, whose mode profile stretches into both branches of the cross, as shown in Fig. S1 (e). No matter which port the input signal is sent, it can be excited. A zoomed-in view shows the field polarization of the Q-mode at the center of cross cavity, which reaches the minimal intensity at the exact center.

Ports 3 and 4 of the cross cavity were used to characterize two distinct cavity modes (D-mode and Q-mode), especially the perpendicular D-mode along the y-axis. When we measured the microwave absorption, ports 3 and 4 of the cross cavity were blocked by two short terminators to prevent weak signal leakages.

SUPPLEMENTARY NOTE 3: TRANSMISSION AND REFLECTION SPECTRUM OF OUR DEVICE

The Hamiltonian of the coupled cavity modes and the magnon mode, as well as their connections with the external photon bath can be constructed as [1, 2]

$$H = H_{sys} + H_{bath} + H_{int} \tag{S6}$$

where H_{sys} represents the Hamiltonian of the intra-cavity system, which contains two types of cavity modes, one magnon mode and the coherent interactions between them. H_{bath} is the Hamiltonian of the extra-cavity photon bath. The H_{int} represents the interaction between two cavity modes and the extra-cavity photon bath. Their detailed expressions are written as:

$$\begin{aligned} H_{sys} &= \hbar\omega_d \hat{d_x}^\dagger \hat{d_x} + \hbar\omega_d \hat{d_y}^\dagger \hat{d_y} + \hbar\omega_m \hat{m}^\dagger \hat{m} + \hbar\omega_q \hat{q}^\dagger \hat{q} + \hbar g_d \sin\theta(\hat{d_x}^\dagger \hat{m} + \hat{d_x}\hat{m}^\dagger) + \hbar g_d \cos\theta(\hat{d_y}^\dagger \hat{m} + \hat{d_y}\hat{m}^\dagger) \\ &\quad + \hbar g_q(\hat{q}^\dagger \hat{m} + \hat{q}\hat{m}^\dagger) \end{aligned} \tag{S7}$$

$$H_{bath} = \int \hbar\omega_k \sum_{r=1}^{4} (\hat{p}^\dagger_{k,r}\hat{p}_{k,r} + \frac{1}{2}) dk \tag{S8}$$

$$\begin{aligned} H_{int} &= \int i\hbar[[\lambda_{d,1}(\hat{p}_{k,1}\hat{d_x}^\dagger - \hat{p}^\dagger_{k,1}\hat{d_x}) + \lambda_{d,2}(\hat{p}_{k,2}\hat{d_x}^\dagger - \hat{p}^\dagger_{k,2}\hat{d_x}) + \lambda_{d,3}(\hat{p}_{k,3}\hat{d_y}^\dagger - \hat{p}^\dagger_{k,3}\hat{d_y}) + \lambda_{d,4}(\hat{p}_{k,4}\hat{d_y}^\dagger - \hat{p}^\dagger_{k,4}\hat{d_y})] \\ &\quad + \sum_{r=1}^{4} \lambda_{q,r}(\hat{p}_{k,r}\hat{q}^\dagger - \hat{p}^\dagger_{k,r}\hat{q})] dk \end{aligned} \tag{S9}$$

where $\hat{d_x}$ ($\hat{d_x}^\dagger$), $\hat{d_y}$ ($\hat{d_y}^\dagger$), $\hat{m}$ ($\hat{m}^\dagger$) and $\hat{q}$ ($\hat{q}^\dagger$) respectively represent the annihilation (creation) operators of the parallel and perpendicular D-modes, the magnon mode and the Q-mode. Correspondingly, ω_d, ω_m and ω_q are their uncoupled mode frequencies. g_d represents the coherent coupling strength between two D-modes and the magnon mode. Considering the field projections onto the spin precession plane [3], effective coupling strengths between two D-modes and the magnon mode are $g_d \sin\theta$ and $g_d \cos\theta$, respectively. The coherent coupling strength between the Q-mode and the magnon mode is weak, and doesn't shows an obviously angular dependency, so that we simply describe it by a constant g_q. $\hat{p}_{k,r}(\hat{p}^\dagger_{k,r})$ represents the annihilation (creation) operator of photons in the photon bath with a wavevector of k at the r-th ($r = 1, 2, 3, 4$) port. For the parallel D-mode $\hat{d_x}$, it couples to ports 1 and 2, while the perpendicular D-mode couples to the ports 3 and 4. Because of the fourfold symmetry of the Q-mode, it couples to all four ports. $\lambda_{d,r}$ and $\lambda_{q,r}$ represent the coupling strengths between cavity modes and the r-th port. They can be independently tuned by adjusting the width of coupling gaps at each port. For the first cross cavity used in our work, four ports are designed to be symmetrical, so that the coupling strengths of four ports are equal and we simply use λ_d and λ_q to represent them.

From Eqs. (S8) and (S9), the input-output relation of the cross cavity can be derived as [2]:

$$p_r^{out} + p_r^{in} = \sqrt{\kappa_d}\hat{d_x} + \sqrt{\kappa_q}e^{i\phi}\hat{q} \tag{S10}$$

Where p_r^{in} and p_r^{out} represent the input and output signals from the r-th port. The term $e^{i\phi}$ is used to describe the initial phase of the Q-mode shifted by the D-mode, which is 0 in the reflection, and π in the transmission.

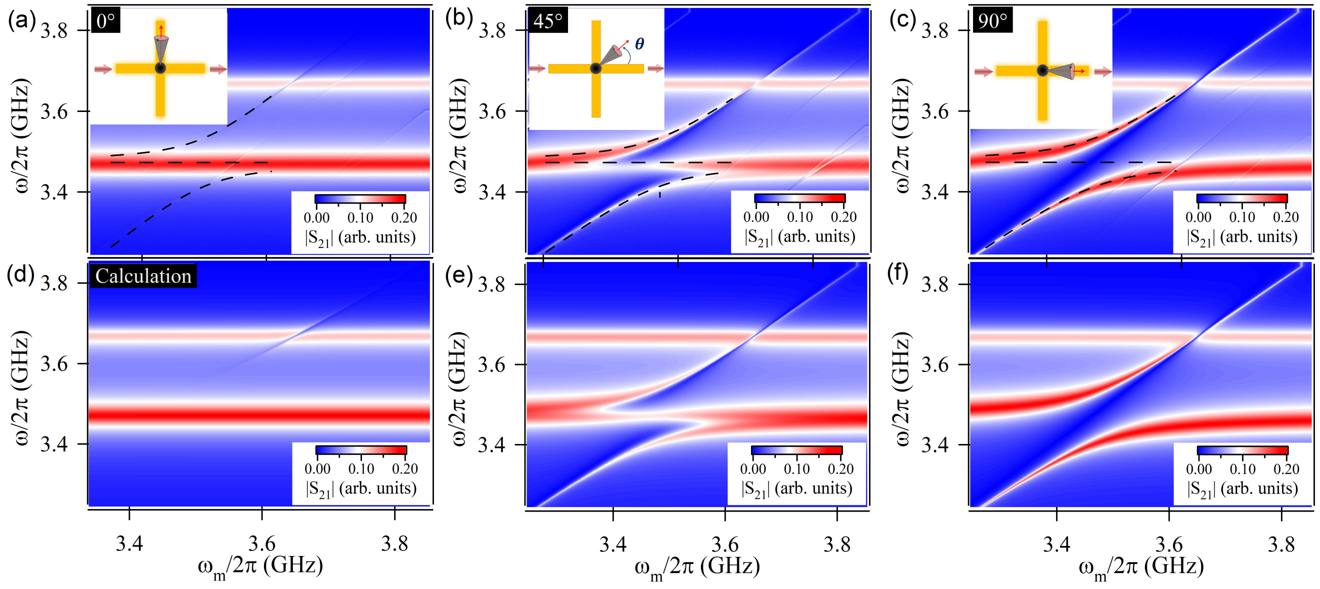

FIG. S3. (a), (b) and (c) Measured transmission mappings of our device at $\theta = 0°$, $45°$ and $90°$, respectively. Black dashed lines in all three figures indicate the calculated dispersions of three CMPs modes arising from the strongly coherent coupling effects between two D-modes and the magnon mode, by using Eq. (S20). (d), (e) and (f) Calculated transmission mappings at three corresponding angles by using Eq. (S17).

The quantum Langevin equation can be derived from Eq. (S7) with considering the interactions between the intra-cavity system and the extra-cavity photon bath:

$$\frac{d}{dt}\begin{bmatrix}\hat{d}_x\\ \hat{d}_y\\ \hat{m}\\ \hat{q}\end{bmatrix} = -i\begin{bmatrix}\omega_d - i(\kappa_d+\gamma_d) & 0 & g_d\sin\theta & 0\\ 0 & \omega_d - i(\kappa_d+\gamma_d) & g_d\cos\theta & 0\\ g_d\sin\theta & g_d\cos\theta & \omega_m - i\gamma_m & g_q\\ 0 & 0 & g_q & \omega_q - i(\kappa_q+\gamma_q)\end{bmatrix}\begin{bmatrix}\hat{d}_x\\ \hat{d}_y\\ \hat{m}\\ \hat{q}\end{bmatrix} + \begin{bmatrix}\sqrt{\kappa_d}\\ 0\\ 0\\ \sqrt{\kappa_q}\end{bmatrix} p_1^{in} \qquad \text{(S11)}$$

Where $\gamma_{d,q}$ and γ_m are the intrinsic damping rates of cavity modes and the magnon mode. Because both the parallel D-mode $\hat{d}_x$ and the Q-mode $\hat{q}$ are connected with the extra-cavity photon bath, an effective dissipative coupling effect generates between them with a strength of $i\sqrt{\kappa_d\kappa_q}$ ($\sim$ 17 MHz). Since this dissipative coupling strength is much smaller than the large detuning between the D-mode and the Q-mode ($\sim$ 200 MHz), we have neglected it during the calculation.

From the first and second rows of Eq. (S11), we can obtain:

$$\hat{d}_x = \frac{ig_d\sin\theta\hat{m} - \sqrt{\kappa_d}p_1^{in}}{i(\omega-\omega_d)-(\kappa_d+\gamma_d)} \qquad \text{(S12)}$$

$$\hat{d}_y = \frac{ig_d\cos\theta\hat{m}}{i(\omega-\omega_d)-(\kappa_d+\gamma_d)} \qquad \text{(S13)}$$

Substituting them into the third row of Eq. (S11), we get:

$$\{[i(\omega-\omega_m)-\gamma_m] + \frac{g_d^2}{i(\omega-\omega_d)-(\kappa_d+\gamma_d)}\}\hat{m} + \frac{ig_d\sin\theta\sqrt{\kappa_d}p_1^{in}}{i(\omega-\omega_d)-(\kappa_d+\gamma_d)} - ig_q\hat{q} = 0 \qquad \text{(S14)}$$

Combining this equation and the fourth row of Eq. (S11), we can obtain the expression of the Q-mode's amplitude:

$$\hat{q} = -\frac{\sqrt{\kappa_q}p_1^{in}}{i(\omega-\omega_q)-(\kappa_q+\gamma_q)+\frac{g_q^2}{i(\omega-\omega_m)-\gamma_m+\frac{g_d^2}{i(\omega-\omega_d)-(\kappa_d+\gamma_d)}}} + \frac{g_d g_q \sin\theta\sqrt{\kappa_d}p_1^{in}}{\{[i(\omega-\omega_m)-\gamma_m][i(\omega-\omega_d)-(\kappa_d+\gamma_d)]+g_d^2\}\{i(\omega-\omega_q)-(\kappa_q+\gamma_q)+\frac{g_q^2}{i(\omega-\omega_m)-\gamma_m+\frac{g_d^2}{i(\omega-\omega_d)-(\kappa_d+\gamma_d)}}\}} \tag{S15}$$

Similarly, we can also derive the amplitude of the parallel D-mode, which is:

$$\hat{d}_x = -\frac{\sqrt{\kappa_d}p_1^{in}}{i(\omega-\omega_d)-(\kappa_d+\gamma_d)+\frac{g_d^2\sin^2\theta}{i(\omega-\omega_m)-\gamma_m+\frac{g_d^2\cos^2\theta}{i(\omega-\omega_d)-(\kappa_d+\gamma_d)}+\frac{g_q^2}{i(\omega-\omega_q)-(\kappa_q+\gamma_q)}}} + \frac{g_d g_q\sin\theta\sqrt{\kappa_q}p_1^{in}}{[i(\omega-\omega_q)-(\kappa_q+\gamma_q)]\{[i(\omega-\omega_d)-(\kappa_d+\gamma_d)][i(\omega-\omega_m)-\gamma_m+\frac{g_d^2\cos^2\theta}{i(\omega-\omega_d)-(\kappa_d+\gamma_d)}+\frac{g_q^2}{i(\omega-\omega_q)-(\kappa_q+\gamma_q)}]+g_d^2\sin^2\theta\}} \tag{S16}$$

In transmission spectrum (S_{21}), the initial phase of Q-mode is shifted by the parallel D-mode with a π. Therefore, the second terms of Eqs. (S15) and (S16) cancel with each other. The expression of transmission spectrum becomes:

$$S_{21} = \frac{p_2^{out}}{p_1^{in}} = \frac{\sqrt{\kappa_d}\hat{d}_x - \sqrt{\kappa_q}\hat{q}}{p_1^{in}} = -\frac{\kappa_d}{i(\omega-\omega_d)-(\kappa_d+\gamma_d)+\frac{g_d^2\sin^2\theta}{i(\omega-\omega_m)-\gamma_m+\frac{g_d^2\cos^2\theta}{i(\omega-\omega_d)-(\kappa_d+\gamma_d)}+\frac{g_q^2}{i(\omega-\omega_q)-(\kappa_q+\gamma_q)}}} + \frac{\kappa_q}{i(\omega-\omega_q)-(\kappa_q+\gamma_q)+\frac{g_q^2}{i(\omega-\omega_m)-\gamma_m+\frac{g_d^2}{i(\omega-\omega_d)-(\kappa_d+\gamma_d)}}} \tag{S17}$$

By using this equation, our experimental observations can be well described. Fig. S3 shows the comparison of the experimental data and calculations. With setting $\mu H = 0$ mT, Eq. (S17) can also describe the transmission spectrum of empty cavity, which has been plotted in Fig. 1 (e) and (f) in the main tex.

Beside the transmission, from our theoretical model, the reflection of system can also be derived. The input-output relation of system (Eq. (S10)) is still valid, but the initial phase of Q-mode (ϕ) is zero in the reflection, instead of the aforementioned π in transmission. Accordingly, substituting Eqs. (S15) and (S16) into the input-output relation (Eq. (S10)), the expression of reflection spectrum of our system can be obtained as:

$$S_{11} = -\frac{p_1^{out}}{p_1^{in}} = 1 - (\sqrt{\kappa_d}\hat{d}_x + \sqrt{\kappa_q}\hat{q})/p_1^{in} \tag{S18}$$

In this case, the second terms of Eqs. (S15) and (S16) superpose with each other, instead of cancelling in the transmission.

SUPPLEMENTARY NOTE 4: INTERACTION BETWEEN TWO D-MODES AND THE MAGNON MODE

If we only consider the coherent coupling effect between two D-modes and the magnon mode, the Q-mode can be neglected, because it's far detuned from the D-mode ($\approx$ 200 MHz) and weakly coupled to the magnon mode. The quantum Langevin equation of the coupled D-modes and magnon mode can be directly obtained from Eq. (S11), which is:

$$\frac{d}{dt}\begin{bmatrix}\hat{d}_x\\ \hat{d}_y\\ \hat{m}\end{bmatrix} = -i\begin{bmatrix}\omega_d - i(\kappa_d+\gamma_d) & 0 & g_d\sin\theta\\ 0 & \omega_d - i(\kappa_d+\gamma_d) & g_d\cos\theta\\ g_d\sin\theta & g_d\cos\theta & \omega_m - i\gamma_m\end{bmatrix}\begin{bmatrix}\hat{d}_x\\ \hat{d}_y\\ \hat{m}\end{bmatrix} + \begin{bmatrix}\sqrt{\kappa_d}\\ 0\\ 0\end{bmatrix}p_1^{in} \tag{S19}$$

From this matrix equation, frequencies of three hybrid modes can be obtained by solving the eigenvalues of the coupling matrix.

$$\widetilde{\omega}_\pm = \frac{1}{2}\left[\omega_d+\omega_m-i(\kappa_d+\gamma_d+\gamma_m)\pm\sqrt{[(\omega_d-\omega_m)-i(\kappa_d+\gamma_d-\gamma_m)]^2+4g_d^2}\right] \quad \textit{and} \quad \widetilde{\omega}_{cen} = \omega_d - i(\kappa_d+\gamma_d) \tag{S20}$$

where, $\widetilde{\omega}_{\pm}$ and $\widetilde{\omega}_{cen}$ respectively represent the upper-branch, lower-branch and central hybrid modes of system.

Without considering the Q-mode, the input-output relation of the coupled D-modes and magnon mode becomes:

$$p_r^{out} + p_r^{in} = \sqrt{\kappa_d}\hat{d}_x \tag{S21}$$

The transmission spectrum of the coupled D-modes and magnon mode can be derived as:

$$\begin{aligned} S_{21} = \frac{p_2^{out}}{p_1^{in}} &= -\frac{\kappa_d}{i(\omega-\omega_d)-(\kappa_d+\gamma_d)+\frac{g_d^2\sin^2\theta}{i(\omega-\omega_m)-\gamma_m+\frac{g_d^2\cos^2\theta}{i(\omega-\omega_d)-(\kappa_d+\gamma_d)}}} \\ &= -\frac{\kappa_d[(\omega-\widetilde{\omega}_m)(\omega-\widetilde{\omega}_d)-g_d^2\sin^2\theta]}{i(\omega-\widetilde{\omega}_d)[(\omega-\widetilde{\omega}_m)(\omega-\widetilde{\omega}_d)-g_d^2]} \\ &= -\frac{\kappa_d[(\omega-\widetilde{\omega}_+)(\omega-\widetilde{\omega}_-)+g_d^2\cos^2\theta]}{i(\omega-\widetilde{\omega}_d)(\omega-\widetilde{\omega}_+)(\omega-\widetilde{\omega}_-)} \\ &= -\frac{\kappa_d}{i(\omega-\widetilde{\omega}_d)}[1+\frac{g_d^2\cos^2\theta}{(\omega-\widetilde{\omega}_+)(\omega-\widetilde{\omega}_-)}] \end{aligned} \tag{S22}$$

From this equation, it's clear see that there exist three maximal values of $|S_{21}|$ at ω_d and $\omega_{\pm}$, respectively.

At zero detuning (i.e., $\omega_m = \omega_d$), $\widetilde{\omega}_{\pm} = \omega_d \pm g_d - i(\kappa_d+\gamma_d+\gamma_m)/2$. When $\omega = \omega_{cen} = \omega_d$, considering $g_d \gg (\kappa_d+\gamma_d+\gamma_m)/2$, the intensity of the central mode (I_c) obtained from Eq. (S22) is:

$$\begin{aligned} I_c \propto |S_{21}|_c &= \frac{\kappa_d}{\kappa_d+\gamma_d}[1-\frac{g_d^2\cos^2\theta}{g_d^2-(\kappa_d+\gamma_d+\gamma_m)^2/4}] \\ &\approx \frac{\kappa_d g_d^2\sin^2\theta}{(\kappa_d+\gamma_d)[g_d^2-(\kappa_d+\gamma_d+\gamma_m)^2/4]} \end{aligned} \tag{S23}$$

The amplitude of central CMP mode follows a $\sin^2\theta$ dependence on θ. By contrast, when $\omega = \omega_{\pm}$, the amplitudes of two side modes ($I_{\pm}$) are:

$$\begin{aligned} I_{\pm} \propto |S_{21}|_{\pm} &= \left| -\frac{\kappa_d}{\pm i g_d - (\kappa_d+\gamma_d)}[1+\frac{g_d^2\cos^2\theta}{\pm 2 i g_d \gamma_{\pm} - \gamma_{\pm}^2}] \right| \\ &\approx \frac{\kappa_d\cos^2\theta}{\kappa_d+\gamma_d+\gamma_m} \end{aligned} \tag{S24}$$

which follows a $\cos^2\theta$ dependence on θ.

SUPPLEMENTARY NOTE 5: COHERENTLY COUPLED PENDULUM SYSTEM WITH TWO DRIVE FORCES

To understand the level attraction arising from the destructive interference, we would use a coupled pendulum system as an example to clarify the physical mechanism. Fig. S4 (a) shows a coupled pendulum system with two drive forces. The dynamic equation of this system can be written as:

$$\begin{bmatrix} \omega-\omega_1+i\gamma_1 & -g \\ -g & \omega-\omega_2+i\gamma_2 \end{bmatrix} \begin{bmatrix} x_1 \\ x_2 \end{bmatrix} = \begin{bmatrix} f_1 \\ f_2 \end{bmatrix} \tag{S25}$$

Here, x_1 and x_2 represent the oscillating amplitudes of two pendulums. γ_1 and γ_2 represent their damping rates, respectively. g is the coupling strength between them, which is a real number. $f_1e^{-i\omega t}$ and $f_2e^{-i\omega t}$ are the two drive forces respectively applied on two pendulums. By solving this matrix equation, we can get the oscillation for each pendulum:

$$\begin{aligned} x_1(\omega) &= \frac{f_1(\omega-\omega_2+i\gamma_2)-gf_2}{[\omega-\omega_1+i\gamma_1][\omega-\omega_2+i\gamma_2]-g^2} \\ x_2(\omega) &= \frac{f_2(\omega-\omega_1+i\gamma_1)-gf_1}{[\omega-\omega_1+i\gamma_1][\omega-\omega_2+i\gamma_2]-g^2} \end{aligned} \tag{S26}$$

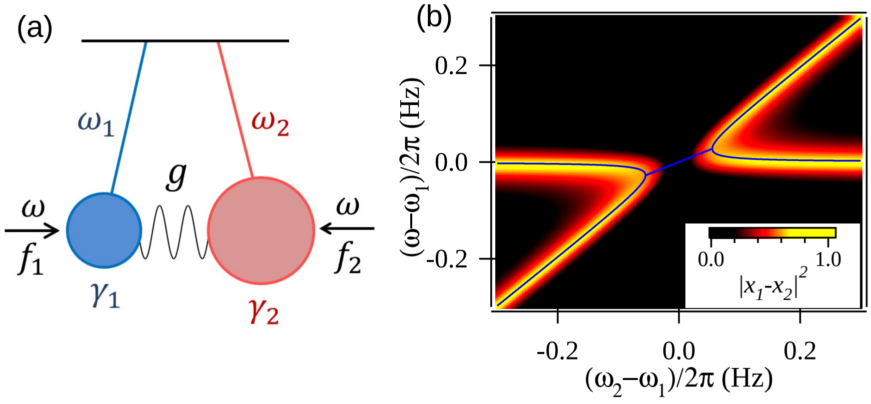

FIG. S4. (a) A schematic picture of coupled pendulums. Two driven forces f_1 and f_2 with a same frequency ω are simultaneously applied on each pendulum. (b) The amplitude of the system response, $R(\omega) \propto |x_1 - x_2|^2$, mapped as a function of detuning $(\omega_2 - \omega_1)$ and ω. Blue solid lines indicate the calculated maximal response of system by using Eq. (S29), which exhibit as a level attraction. Here we use $\gamma_1/2\pi = \gamma_2/2\pi$=0.03 Hz $g/2\pi$=0.012 Hz and $f_2 = f_1$.

Considering that both pendulums are connected with external drives, the response function of system should be a combination of oscillations from each pendulum. From Eq. (S26), we can get:

$$x_1(\omega) - x_2(\omega) = \frac{f_1(\omega - \omega_2 + i\gamma_2) - f_2(\omega - \omega_1 + i\gamma_1) + g(f_1 - f_2)}{[\omega - \omega_1 + i\gamma_1][\omega - \omega_2 + i\gamma_2] - g^2} \tag{S27}$$

We define the response function as $R(\omega) \propto |x_1 - x_2|^2$ corresponding to 180°-out-of-phase motion between x_1 and x_2. The calculated response function is shown in Fig. S5 (b) with $\omega_1/2\pi = 3.5$ Hz, $\gamma_1/2\pi = \gamma_2/2\pi = 0.03$ Hz, $g/2\pi = 0.012$ Hz and $f_1 = f_2$.

The maximal amplitudes of the response function can be derived from the differential equation $d|R(\omega)|/d\omega = 0$. In the case of $f_1 = f_2$ and $\gamma_1 = \gamma_2$, a cubic equation of ω can be obtained, which has a form of:

$$(2\omega - \omega_1 - \omega_2)[(\omega - \omega_1)(\omega - \omega_2) - g^2 + \gamma_1\gamma_2] = 0 \tag{S28}$$

By solving this equation, two maximal response occurs at:

$$\omega_\pm = \frac{1}{2}(\omega_1 + \omega_2) \pm \frac{1}{2}\mathfrak{Re}[\sqrt{(\omega_1 - \omega_2)^2 + 4(g^2 - \gamma_1\gamma_2)}] \tag{S29}$$

According to this model, $\omega_\pm$ follows a typical level attraction dispersion as long as the dissipation of the system dominates the coherent coupling, i.e., $\gamma_1\gamma_2 > g^2$.

SUPPLEMENTARY NOTE 6: LEVEL ATTRACTION BETWEEN THE Q-MODE AND THE MAGNON MODE

From Eq. (S11), we get:

$$\{[i(\omega - \omega_m) - \gamma_m][i(\omega - \omega_d) - (\kappa_d + \gamma_d)] + g_d^2\}\hat{m} - ig_q[i(\omega - \omega_d) - (\kappa_d + \gamma_d)]\hat{q} = -ig_d \sin\theta\sqrt{\kappa_d}p_1^{in} \tag{S30}$$

We note that the coefficient before the magnon operator $\hat{m}$ can be simplified into a much simpler form than it present by substituting the eigenvalues of the up-branch and lower-branch hybridized modes $(\widetilde{\omega}_\pm)$. Therefore, we get:

$$[i(\omega - \widetilde{\omega}_+)][i(\omega - \widetilde{\omega}_-)]\hat{m} - ig_q[i(\omega - \omega_d) - (\kappa_d + \gamma_d)]\hat{q} = -ig_d \sin\theta\sqrt{\kappa_d}p_1^{in}$$
$$i(\omega - \widetilde{\omega}_+)\hat{m} - ig_q\frac{i(\omega - \omega_d) - (\kappa_d + \gamma_d)}{i(\omega - \widetilde{\omega}_-)}\hat{q} = \frac{-ig_d \sin\theta\sqrt{\kappa_d}p_1^{in}}{i(\omega - \widetilde{\omega}_-)} \tag{S31}$$

When the magnon mode gets close to the Q-mode, the lower-branch hybridized mode $(\widetilde{\omega}_-)$ returns to the frequency of D-modes, i.e., $\omega_- \approx \omega_d$. Therefore, we can get $i(\omega - \omega_d) - (\kappa_d + \gamma_d) \approx i(\omega - \widetilde{\omega}_-)$. Then the Eq. (S31) becomes:

$$i(\omega - \widetilde{\omega}_+)\hat{m} - ig_q\hat{q} = \frac{-ig_d \sin\theta\sqrt{\kappa_d}p_1^{in}}{i(\omega - \widetilde{\omega}_-)} \tag{S32}$$

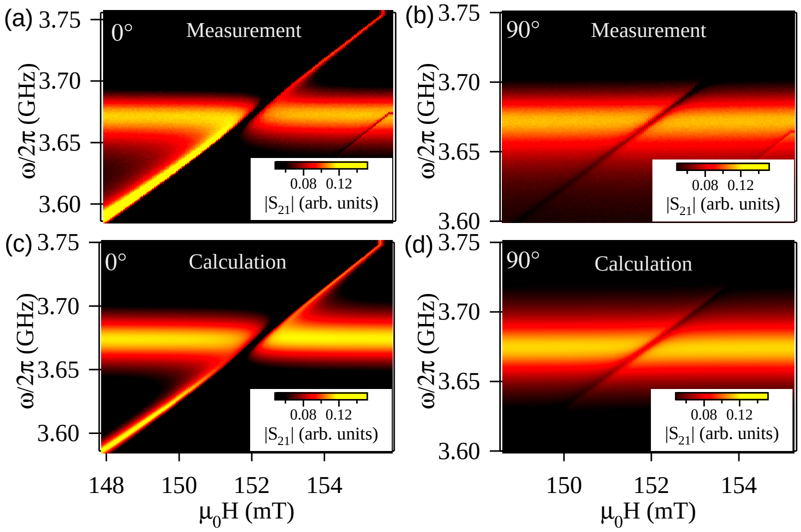

FIG. S5. (a) and (b) Measured and (c) and (d) calculated transmission spectra $|S_{21}|$ are plotted as a function of external magnetic field (H) and frequency (ω). As the direction of the external magnetic field is rotated from 0° to 90°, the mode hybridization behaviour between the magnon mode and the Q-mode changes from level repulsion to level attraction.

Combining the 4th row of Eq. (S11) and Eq. (S32), we get a 2×2 matrix:

$$\begin{bmatrix} \omega - \widetilde{\omega}_+ & -g_q \\ -g_q & \omega - \widetilde{\omega}_q \end{bmatrix} \begin{bmatrix} \hat{m} \\ \hat{q} \end{bmatrix} = \begin{bmatrix} i\frac{g_d \sin\theta\sqrt{\kappa_d}}{(\omega - \widetilde{\omega}_-)} \\ i\sqrt{\kappa_q} \end{bmatrix} p_1^{in} \tag{S33}$$

where $\widetilde{\omega}_q$ is the complex frequency of the Q-mode, i.e., $\widetilde{\omega}_q = \omega_q - i(\kappa_q + \gamma_q)$. From this equation, we note that: (i) the interaction between the cavity Q-mode and the magnon mode is still coherent, and the coupling strength is g_q; (ii) different from usual cavity magnon polariton systems, in this case, the input signal p_1^{in} simultaneously drives the Q-mode and the magnon mode.

From Eq. (S33), we assume an equivalent coupling strength between the extra-cavity photon bath and intra-cavity magnon mode as κ_+, which has a form of:

$$\kappa_+ = \frac{g_d^2 \sin^2\theta}{(\omega - \widetilde{\omega}_-)^2}\kappa_d \tag{S34}$$

Therefore, an effective input-output relation for the coupled Q-mode and the magnon mode can be written as:

$$p_r^{out} + p_r^{in} \approx \sqrt{\kappa_+}\hat{m} + \sqrt{\kappa_q}e^{i\phi}\hat{q} \tag{S35}$$

By setting $\phi = \pi$, the transmission spectrum can be solved as:

$$\begin{aligned} S_{21} = \frac{p_2^{out}}{p_1^{in}} &\approx -\frac{\kappa_+}{i(\omega - \widetilde{\omega}_+) + \frac{g_q^2}{i(\omega - \widetilde{\omega}_q)}} + \frac{\kappa_q}{i(\omega - \widetilde{\omega}_q) + \frac{g_q^2}{i(\omega - \widetilde{\omega}_+)}} \\ &= \frac{-i\kappa_+(\omega - \widetilde{\omega}_q) + i\kappa_q(\omega - \widetilde{\omega}_+)}{[i(\omega - \widetilde{\omega}_q)][i(\omega - \widetilde{\omega}_+)] + g_q^2} \end{aligned} \tag{S36}$$

Calculated mappings by using this equation have been plotted in Fig. S5 (c) and (d), which can well reproduce the modes hybridization behaviours between the magnon mode and the Q-mode in the transmission.

To further understand the level attraction in our device, three typical transmission spectra are extracted from Fig. S5 (b), and plotted in the Fig. S6. In the amplitude spectra (Fig. S6 (a)-(c)), the green and red arrows respectively indicate the cavity-like and magnon-like modes, while the blue arrow indicates the anti-resonant dip which follows the dispersion of the uncoupled magnon mode. Both hybrid modes (resonant peaks) lie at one side of the anti-resonant dip. When the magnon mode frequency is tuned to match with the Q-mode, i.e., the zero-detuning condition ($\mu_0 H = 151.2$

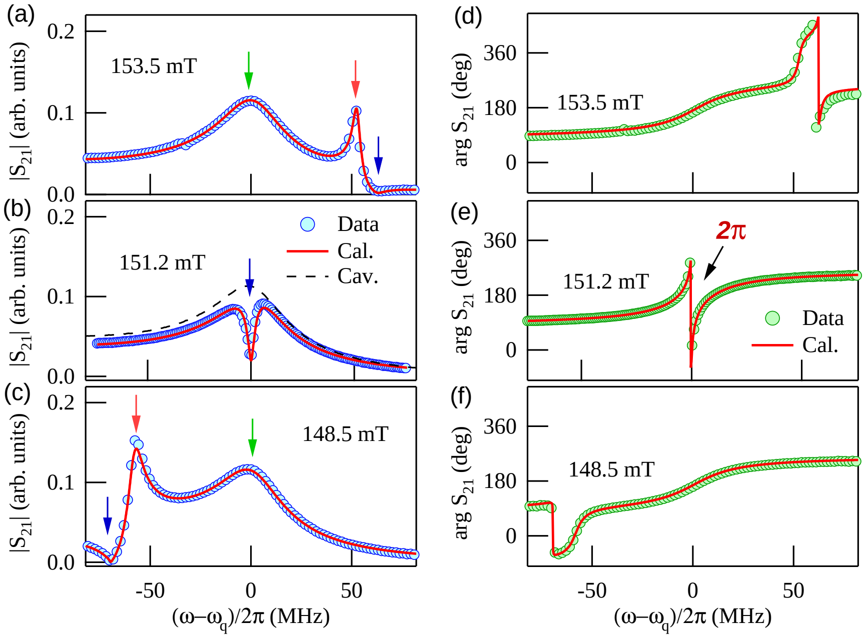

FIG. S6. (a)-(c) Amplitude of three typical spectra. Green and red arrows respectively indicate the cavity-like and magnon-like hybrid modes in the coupled Q-mode and magnon mode system. Blue arrow indicates the anti-resonant dip. Dots and red solid lines are experimental data and calculation results (Eq. S17 which has no approximation). Black dashed line in (b) is the transmission of the bare Q-mode. (d)-(f) Phase spectra of these three transmission spectra. At the zero detuning, $\mu_0 H = 151.2$ mT, a sharp 2π-phase jump occurs.

mT), a sharp dip occurs which exhibits a magnetically induced transparency (MIT)-like spectra. Correspondingly, phase evolutions of these three spectra are plotted in Fig. S6 (d)-(f). As all level attraction cases reported in previous works, a sharp 2π-phase jump occurs, especially at the zero-detuning ($\mu_0 H = 151.2$ mT), which has been recognized as a fingerprint of the level attraction behaviour. Red solid lines in Fig. S6 represent the calculation results, which well reproduce our experimental observations.

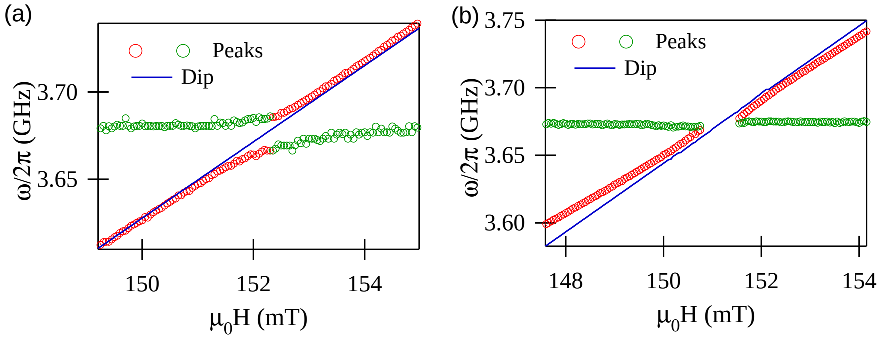

FIG. S7. (a) The dispersions of the cavity-like (green dots) and magnon-like (red dots) modes readout from the Fig. S5 (a). Blue solid line indicates the antiresonance of the uncoupled magnon mode. (b) The dispersions of two hybrid modes and one anti-resonance readout from the Fig. S5 (b).

The frequencies of two hybridized modes and one anti-resonant dip in Fig. S5 (a) and (b) have been read out and plotted in Fig. S7. Green and blue dots respectively indicate the cavity-like hybridized mode and the magnon-like hybridized mode, while the blue solid line in both figures indicate the frequencies of anti-resonant dips which follows the dispersion of the uncoupled magnon mode. Two hybridized modes in Fig. S7 (a) respectively occur at two sides of the antiresonance, so that a level repulsion can be observed. On the contrary, two hybridized modes in Fig. S7 (b)

coalesce at one side of the antiresonance (blue solid line) and exhibit a typical level attraction behaviour.

SUPPLEMENTARY NOTE 7: MIPA CONDITION

Following Eq. (S33) we can obtain the expressions for $\sqrt{\kappa_+}\hat{m}$ and $\sqrt{\kappa_q}\hat{q}$ as,

$$\sqrt{\kappa_+}\hat{m} = \frac{-i(iIm(\tilde{\omega}_q)\kappa_+ + g_q\sqrt{\kappa_+\kappa_q} + \kappa_+(\omega-\omega_q))}{g_q^2 + (-iIm(\tilde{\omega}_+)-\omega+\omega_1)(iIm(\tilde{\omega}_q)+\omega-\omega_q)} \quad \text{(S37)}$$

$$\sqrt{\kappa_q}\hat{q} = \frac{-i(g_q\sqrt{\kappa_+\kappa_q} + \kappa_q(iIm(\tilde{\omega}_+)+\omega-\omega_+)))}{g_q^2 + (-iIm(\tilde{\omega}_+)-\omega+\omega_1)(iIm(\tilde{\omega}_q)+\omega-\omega_q)}. \quad \text{(S38)}$$

where ω_+ and $Im(\tilde{\omega}_+)$ are the real and imaginary parts of $\tilde{\omega}_+$, ω_q and $Im(\tilde{\omega}_q)$ are the real and imaginary parts of $\tilde{\omega}_q$. When $\sqrt{\kappa_+}\hat{m}$ and $\sqrt{\kappa_q}\hat{q}$ equals to 1/2, the perfect absorption condition is satisfied. In this manner, we can obtain that

$$-Im(\tilde{\omega}_+)Im(\tilde{\omega}_q) - g_q^2 + 2Im(\tilde{\omega}_q)\kappa_+ + (\omega-\omega_1)(\omega-\omega_q) = 0, \quad \text{(S39)}$$

$$-Im(\tilde{\omega}_+)Im(\tilde{\omega}_q) - g_q^2 + 2Im(\tilde{\omega}_+)\kappa_q + (\omega-\omega_1)(\omega-\omega_q) = 0. \quad \text{(S40)}$$

By neglecting the small quantity of $(\omega-\omega_+)^2$, we get the condition of $\kappa_+\kappa_q \approx g_q^2 + \gamma_+\gamma_q - (\omega_+ - \omega_q)(\omega - \omega_+)$.

SUPPLEMENTARY NOTE 8: PARAMETERS OF THE MODIFIED CROSS CAVITY

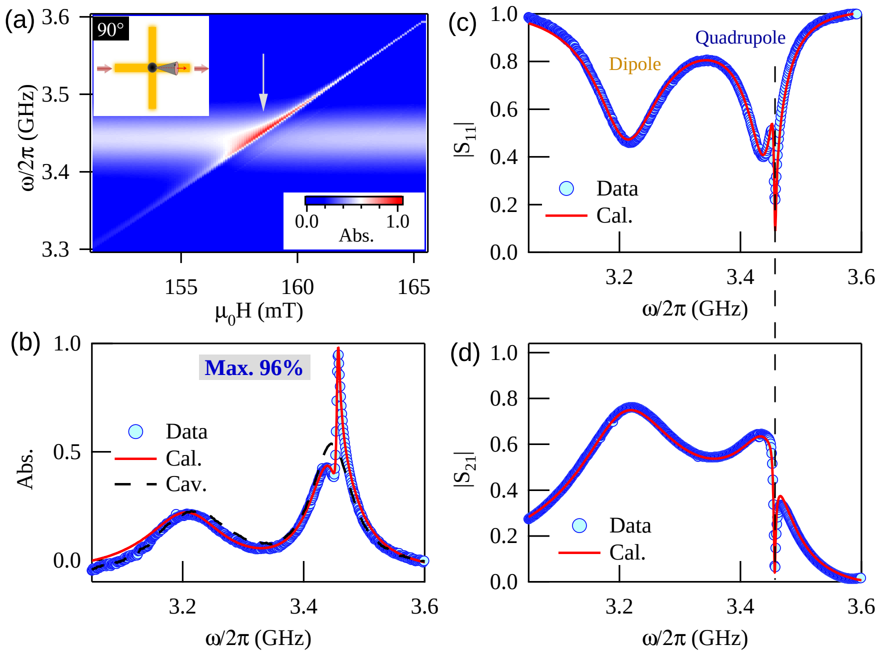

FIG. S8. (a) Measured absorption rate of the modified cross cavity at $\theta = 90°$. When the magnon mode approaches the Q-mode, the nearly perfect absorption of the input signal occurs. (b) Absorption spectrum measured at 158 mT (i.e., $\omega_m/2\pi = 3.449$ GHz). The black dashed line indicates the measured absorption of the empty cavity. (c) and (d) Reflection and transmission spectrum at 158 mT. Blue dots and red solid lines respectively represent the experimental data and calculation results.

According to our theoretical prediction, we have fabricated another cross cavity, whose coupling gaps of ports are much narrower than the former one, with only 50 μm. Parameters of this modified cross cavity are listed in Tab. 1.

TABLE I. Mode frequencies, extrinsic damping rates, intrinsic damping rates of the D- and Q-modes

D-mode	$\omega_d/2\pi = 3.216$ GHz	$\gamma_d/2\pi = 17$ MHz	$\kappa_{d1}/2\pi = 44.7$ MHz	$\kappa_{d2}/2\pi = 84$ MHz
Q-mode	$\omega_q/2\pi = 3.448$ GHz	$\gamma_q/2\pi = 16$ MHz	$\kappa_{q1}/2\pi = 28.5$ MHz	$\kappa_{q2}/2\pi = 22.5$ MHz

The coupling strength between two D-modes and the magnon mode is $g_d/2\pi = 42$ MHz, while the coupling strength between the Q-mode and the magnon mode is $g_q/2\pi = 4$ MHz. We note that the two ports of this new cross cavity are a little asymmetric, especially for the D-mode i.e., $\kappa_{d1} \neq \kappa_{d2}$, but our theoretical model is still valid. Red solid lines in Fig. S8 are the calculation results.

[1] J. Rao, S. Kaur, B. Yao, E. R. J. Edwards, Y. Zhao, X. Fan, D. Xue, T. J. Silva, Y. Gui and C. -M. Hu, Nat. Commun. **10**, 2934 (2019).

[2] D. F. Walls and G. J. Milburn, Quantum Optics, Springer-Verlag, Berlin, (1994).

[3] Lihui Bai, Michael Harder, Paul Hyde, Zhaohui Zhang, Can-Ming Hu, Y.P. Chen and John Q. Xiao, Phys. Rev. Lett. **118**, 217201 (2017).
